# Medical News and Miscellany

**Published:** 1888-11

**Authors:** 


### MEDICAL NEWS AND MISCELLANY.

THE TEXAS QUARANTINE, at Texarkana, has just been raised.

DR. A. W. CRAWFORD, of Midlothian, is convalescent from a severe spell of sickness.

DR. J. W. T. STEVENS, of Mt. Calm, has just recovered from a spell of typhoid fever.

DR. D. B. MCGEE, of Hinckley, is attending a special course at Tulane University, medical department., New Orleans.

MARRIED—At Houston, Texas. November 7, Mr. John Hagemann to Miss Nettie Burke Daniel, daughter of Dr. J. W. Daniel, of that city.

DR. C. T. COOPER, of Lilac, Milam county, is attending the clinics of the Chicago Medical College, and directs his JOURNAL forwarded to hin.

THE SOUTHERN SURGICAL AND GYNECOLOGICAL ASSOCIATION will hold its annual meeting in Birmingham, Alabama, December 4, 5 and 6, prox.

DR. J. A. SOUTHWORTH, of Corsicana, had the misfortune to lose his youngest child, September 25, a bright little boy. We tender our sympathy to the doctor.

FOR SALE.—$2,500 or $3,000 practice, with residence, for sale, in healthy location. City of 2500 inhabitants. Price of residence $2,500. Terms reasonable. Address this office, L.

MARRIED—October 27th ult., Dr. J. Dixon Bruns, of New

Orleans, late editor-in-chief of the *N. O. M. & S. Journal*, to Miss Kate Logan, daughter of General and Mrs. Thos. M. Logan, of Virginia.

---

DR. W. F. BLOUNT, late the efficient and popular quarantine officer at Galveston, having resigned, has removed to Lockhart and resumed the practice of medicine.

---

AND STILL THEY COME.—Drs. Legrande & Bowcock will, on the 15th of December prox., issue at Anniston, Ala., a medical journal, to be called the *Alabama Medical and Surgical Age*. Success to the chick.

---

A DOCTOR DISSECTS a live subject, and is himself dissected, in Alabama. A Dr. Nabors, of Montevallo, had a difficulty with a lawyer Shortridge, and they fought a duel with bowie knives in a dark room. Result,—two cadavers in a bad state of mutilation.

---

THE AMERICAN PUBLIC HEALTH ASSOCIATION will meet in Milwaukee, Wisc., November 20, 21, 22 and 23 (inst.) We observe, amongst the papers to be read, is one "The History and Administration of Quarantine in Texas 1878 to 1888; ny R. Rutherford, M. D., State Health Officer of Texas.

---

THE NEW ORLEANS POLYCLINIC.—This institution, with a full corps of able instructors has been successfully inaugurated. The spring session will open on the first Monday in April, 1889, and continue two full months. The great Charity Hospital affords abundance of clinical material. See announcement in our advertisement pages—and address Dr. J. H. Bemiss, President, for particulars.

---

DEATH OF MRS. PATTEN.—We are pained to announce the death of Mrs. E. E. Patten, wife of Dr. A. L. Patten, and mother of Dr. A. Patten, both of Mineola, Texas. Mrs. Patten, for more than thirty years, had resided in Wood county, where she was always a ministering angel of kindness and charity, seeking ever to mitigate the burdens and sorrows of the poor and afflicted. She died at Mineola, Sunday morning, October 28, after an illness of two weeks.

---

REMOVAL.—Dr. S. H. Weatherford has removed from Corn Hill to Georgetown, Williamson county.

---

CHARLES H. PHILLIPS, for many years a prominent manufacturer of chemicals in New York, and president of the enterprising and well known "Chas. H. Phillips Chemical Co.," 30 Platt street, New York, died suddenly, of apoplexy, last week. His loss will be seriously felt in commercial and social circles.

---

PARK, DAVIS & CO., the enterprising manufacturers of pharmaceuticals, has "downed" a would be monopolist of the name of acid phosphate, and before the courts of the country has demonstrated that one man has as much right to manufacture and sell phosphoric acid as another, however rich and bull-headed. See fourth cover of our August number.

---

PROF. OF MATERIA MEDICA, (lecturing on tannin)—"And by the bye, gentlemen, tannic acid is the antidote to the poison of mushroom; can any of you explain its action?"

Texas Student—"T-t-think I can, professor!"

"Well, sir, explain to the class the chemical reactions that occur, and how tannin acts as antidote to the poison of the poisonous mushroom."

"It f-f-forms the t-t-tannate of m-mush, and leaves room in the s-s-stomach!

---

AS WAS to be expected, our denunciation of the hog as an article of human food, has attracted attention, and has elicited several articles in reply, only one, however, intended for publication. Dr. J. L. Cunningham, of Dallas, a venerable and well-known practitioner of Texas, takes up the cudgel in defense of the hog and denies our allegations that the hog is unclean, or filthy in his habits. But we anticipate ; the article will appear in full in our next issue, and no doubt will be much relished by all lovers of "chine and spare ribs" as the doctor says. Dr. Cunningham sent along $2 at same time to help the cause of journalism.

---

"THE FLOWERS THAT BLOOM IN THE SPRING, TRA-LA."—An eminent medical man remarked in our presence once, "Literature and the love of the beantiful go hand in hand." We believe that most educated physicians love flowers, and children. We pity the man who is not touched by their beauty and innocence.

If you would have the delights of early spring flowers—the modest and spotless lily, the gay tulips, the fragrant hyacinth and the Poet's flower, narcissus; crocusses, and snow drops, tube roses, and the stately and elegant amarylis, as well as roses to bloom early and often, send *now* for them. We have just finished planting a large lot, bought of GOOD & REESE, Springfield, Ohio. They sell at half the price charged by many other well-known dealers and send roses and other plants *double* the size; two years old, well rooted roses, *named*, and of the best varieties for 15 cents! and *twenty named roses*, perpetual bloomers, smaller but well rooted, for *one dollar !* and all other standard plants and bulbs in proportion. Our plants and bulbs, a large lot, came through in splendid shape, green and fresh, and the plants went to growing, and some of them, to *blooming*, right away! Mention this JOURNAL.

---

P. M. B. A.—The Circular Letter from the Board of Directors of the P. M. B. A. of Texas, to the profession, will appear in next issue. Look for it.

